# Genetic Diversity of PRRS Virus Collected from Air Samples in Four Different Regions of Concentrated Swine Production during a High Incidence Season

**DOI:** 10.3390/v6114424

**Published:** 2014-11-14

**Authors:** Barbara Brito, Scott Dee, Spencer Wayne, Julio Alvarez, Andres Perez

**Affiliations:** 1 Department of Veterinary Population Medicine, College of Veterinary Medicine, University of Minnesota, 385A Animal Science Veterinary Medicine Building, 1988 Fitch Avenue, St. Paul, MN 55108, USA; E-Mails: jalvarez@umn.edu (J.A.), aperez@umn.edu (A.P.); 2 Pipestone Veterinary Services, Pipestone, MN 56164, USA; E-Mails: sdee@pipevet.com (S.D.); swayne@pipevet.com (S.W.)

**Keywords:** porcine, Porcine Reproductive and Respiratory Syndrome, virus, livestock diseases, air filtration, Molecular Epidemiology

## Abstract

Porcine Reproductive and Respiratory Syndrome (PRRS) is one of the most relevant swine diseases in the US, costing the industry millions of dollars per year. Unfortunately, disease control is difficult because of the virus dynamics, as PRRS virus (PRRSV) can be transmitted by air between farms, especially, in regions with high density of swine operations. While long distance airborne transport of PRRSV has been reported, there is little information regarding the dynamics of PRRSV airborne challenge in concentrated regions. The objective of this study was to describe the frequency of detection, dose and diversity of PRRSV in air samples collected across four concentrated production regions during the PRRS-high risk season in the Midwestern US (October–December) in 2012. Between 29% and 42% of the air samples were positive in all four sampling sites. Sequencing of the recovered virus showed a wide diversity of field and vaccine variants. Higher frequency, dose, and diversity of PRRSV were observed in air at locations with higher pig density. These findings suggest that regional spread of PRRSV due to aerosol transmission of PRRSV represents a significant risk to susceptible herds in concentrated regions of domestic pig production where PRRSV is endemic.

## 1. Introduction

Porcine Reproductive and Respiratory Syndrome (PRRS) virus (PRRSV) was first identified in 1991, and is now present in many countries worldwide [1,2] PRRSV is one of the primary causes of decreased swine production with losses estimated at 664 million dollars per year to the US industry [3]. PRRSV is a member of the genus *Arterivirus* [4], a single stranded positive sense enveloped RNA virus with a genome containing nine open reading frames (ORF) that code for seven structural proteins and 14 non-structural proteins [5].

Control and elimination of PRRSV in the US is challenging due to the high concentration of swine production in specific areas of the country along with an absence of official regulation, such as reporting or mandatory control actions [6]. Another aspect complicating PRRS control is the ability of the virus to be transported over extended distances by contaminated aerosols [7,8,9]. While data exist regarding the ability of the virus be transported via aerosols up to 9.2 m [9], little is known about the dynamics of daily PRRSV airborne challenge directly at the farm level. If the level of airborne PRRSV challenge on a day-to-day basis could be documented, decisions regarding the application of intervention strategies capable of reducing the airborne risk (e.g., area vaccination or air filtration) would be easier to make [10,11].

Therefore, in order to document the risk of PRRSV airborne challenge, the objective of this study was to measure frequency of detection and the dose and the diversity of PRRSV observed in daily air samples collected around four regions that differed in farm and pig density. Novel techniques were employed, including liquid cyclonic collectors for recovering virus from air [12] and Bayesian phylogenetic analysis of sequenced virus [13]. Characterization of virus detected in air samples will help guiding decisions regarding biosecurity measures in swine farms.

## 2. Materials and Methods

### 2.1. Air Sampling Locations

Air samples were collected in the surroundings of four commercial sow units. The study farms were located in Eastern South Dakota (farm A), Northeast Iowa (farm B), Southwest Minnesota (farm C), and Northwest Iowa (farm D). Approximately 90% of the neighboring farms were finishers or wean-to finish type. County pig density was obtained from the National Agricultural Statistics Service census of Agriculture statistics 2012 census [14]. The four sow farms have similar, strict biosecurity protocols, including air filtration and introduction of gilts from PRRS-negative multipliers. Three of the four farms had no PRRS positive animals during the study period. In location D, three animal samples were positive to vaccine strain virus, and one to RFLP 1-26-2 virus.

To visualize the farm density in each of the four locations, neighboring pig farms within a 10 km radius, were mapped using a kernel density, implemented in an open source GIS software [15]. The radius set for the kernel density (influence of a farm in a circular area) was set to 5 km, using a quadratic bi-weight kernel shape.

### 2.2. Air Sample Collection and Quality Control Methods

For the purpose of air collection, a sampling kit was sent to each of the four study farms. The kit consisted of a plastic tote containing a liquid cyclonic collector (Midwest MicroTek, Brooking, SD, USA), sterile 7.2% saline (Nova-Tech, Grand Island, NE, USA), a notebook for record keeping, permanent markers, individually wrapped sterile Dacron swabs (Fisher Scientific, Waltham, MA, USA), 3 mL plastic Falcon tubes (Becton Dickinson, Franklin Lakes, NJ, USA), a box of disposable gloves, a spray bottle of disinfectant (0.8% Synergize, Preserve International, Atlanta, GA, USA) and paper towels.

For the purpose of quality control, prior to shipping to the farm, each item in the kit, along with its plastic container was swabbed and tested for the presence of PRRSV RNA by PCR. From October 15 through December 15, 2012, a period of time described as the peak epidemic season for PRRS in the region [16], daily air samples were collected outside of the facility from 7–7:30 AM each day. A liquid cyclonic collector was placed 30 m from the facility in the direction of the daily prevailing wind and samples were collected as described elsewhere [12]. To maximize consistency and quality of the sampling process, a designated individual on each farm conducted the sampling and handled all air samples. Those four individuals had been trained in using the sampling equipment, the proper handling of samples and record keeping using validated protocols. Following each daily collection, samples were stored on farm in an external freezer at −20 °C and the collection equipment was sanitized and swabbed to document the absence of residual PRRSV RNA. Finally, to validate the specificity of the sampling process, air samples were collected on an abandoned farm site. This site housed no swine and the closest swine farm to this site was 15 km away. For the purpose of air collection, a wall fan from an abandoned building was turned on for 30 minutes and a sample collected. Ten such samples were collected in October, November and December 2012 for a total of 30 samples. Following completion of the project period, all air samples from all farms were sent to the University of Minnesota Veterinary Diagnostic Laboratory (UMN-VDL) for testing.

Should positive samples be detected, it was planned to re-test all PCR positive samples and to re-sequence a subset (25%) of the samples to insure accuracy of laboratory methods. Finally, to insure that accidental sample contamination, secondary to contact with serum or oral fluids samples did not occur at the farm, at the Pipestone Veterinary Clinic (PVC), or at the UMN-VDL, all samples were tested for PRRSV antibodies by ELISA [17].

### 2.3. Polymerase Chain Reaction

All samples collected during the study were tested for the presence of PRRSV RNA using a one‑step TaqMan PCR assay (Perkin-Elmer Applied Biosystems, Foster City, CA, USA) at the UMN‑VDL. The PCR assay detected the ORF 6 region of the North American (NA) PRRSV and the ORF 7 region of the European (EU) PRRSV [18]. Primers and probes employed were as follows:

PRRS NA/EU primers:

- NA F (ORF 6) Forward primer (26 oligomer) (5’⇒3’: sequence: GTAGTYGCRCTCCTTTGGGGGGTGTA)
- NA F2 (ORF 6) Forward primer (33 oligomer) (5’⇒3’: sequence: TTCATCACYTCCAGRTGCCGTTTGTGCYTGCTA)
- NA R (ORF 6) Reverse primer (18 oligomer) (5’⇒3’: sequence: CGASAAATGCGTGGTTAT)
- EU (ORF 7) Forward primer (19 oligomer) (5’⇒3’: sequence: TGGCCAGCCAGT AATCAA)
- EU (ORF 7) Reverse primer (21 oligomer) (5’⇒3’: sequence: TGTGGCTTCTCAGGCTTTTTC)

PRRS NA/EU FAM primers labeled ORF6 TAMRA probe (22 oligomer)

- (5’⇒3’: FAM-TACATTCTGGCCCCTGCCCAYC-TAMRA)

PRRS EU FAM labeled ORF7 TAMRA probe (25 oligomer)

b. (5’⇒3’: 6FAM-TGCAATGATAAAGTCCCAGCGCCAG-TAMRA).

### 2.4. Virus Titration

All PCR-positive air samples were titrated using Marc-145 cells and MEM supplemented with 8% fetal calf serum, antibiotics and antifungal agents, to quantify the level of infectious virus in the sample [19,20].

### 2.5. Sequencing

To estimate genetic diversity of PRRSVs recovered from PCR-positive air samples to those collected from field cases, the ORF 5 region of the PRRSV was sequenced using previously published techniques [21].

### 2.6. Phylogenetic Analysis 

Sequences collected during this study, in addition to 105 sequences available from PVC records of PRRS-infected animals collected from October 15 to December 15, 2012 (study period) were also included. Furthermore 216 sequences from the same database including different viruses collected from 2002 to 2013 were added to increase the robustness of the phylogeny reconstruction. 

All sequences were aligned using MUSCLE [22]. The nucleotide substitution and codon partition schemes were selected using the PartitionFinder software package [23]. The best partition scheme and nucleotide substitution model were selected based on the Bayesian information criteria (BIC). We reconstructed the phylogeny of the sequences by simulation and sampling of trees using a Markov chain Monte Carlo MCMC algorithm implemented in BEAST v1.8 [24]. We compared relaxed uncorrelated exponential and uncorrelated lognormal clock models. For the tree prior we compared the coalescent constant population size and the coalescent Bayesian skyline.

We simulated the trees for 500,000,000 generations and checked that the effective sample size value reached >100 in all parameters. The convergence and mixing of estimates of parameters generated in the simulations were visualized and evaluated in Tracer v1.6 [25]. To determine if a strict clock would be an appropriate assumption for the data instead of the relaxed clock, the distribution of the estimated coefficient of variation of the relaxed models was examined, if 90% of the observations did not include 0, the strict model was ruled out. All four models were run (each relaxed clock with either coalescent constant or Bayesian skyline population). The final model was selected using the posterior simulation-based analogue of Akaike’s information criterion (AICM), which we run 100 times (AICM) [26].

To visualize the tree file generated, the tree was annotated burning all simulations before reaching convergence according to the observed trace. The tree was visualized using Figtree v.1.5 [27].

### 2.7. Diversity of Sequences Found in the Sampling Areas

We compared two approaches to characterize the diversity of PRRSV sampled from air. One of the measures was the traditionally used pairwise percentage of genetic identity and the phylogenetic distance estimated by the maximum clade credibility (MCC) tree estimated by Bayesian methods previously described. For this purpose we computed the sequence identity between all pairs of sequences (number of identical positions divided by the total length of the alignment) using BioEdit [28]. The phylogenetic distance estimated by Bayesian methods was measured as the cophenetic distance between all sequences pairs of the MCC, which was obtained using ape package in R. [29]. The distance between the sequences in this phylogenetic tree, reflect the sum of time of evolution to a common ancestor of each pair of sequences, being a measure that takes into account the estimated relationship of the virus based on its divergence in time. On the other hand, the pairwise percentage of identity only indicates the number of nucleotides in which two aligned sequences differ. The correlation between the percentage identity and cophenetic distance values was computed using Pearson’s coefficient (R), to evaluate the association between those two genetic distance methods.

Histograms of pairwise genetic differences obtained by the two methods were constructed for sequences collected at each air sampling location.

We further classified different viruses as defined by the phylogenetic tree clades. A clade was defined as a group of viruses reaching a common ancestor in 2009 or earlier in time. Summary statistics of sequences identities for each of the clades were also computed.

For each of the sample locations, the frequency of RNA recovery (positive samples divided by total samples collected), and the amount of virus (in TCID_50_/mL) were recorded. Locations were compared in terms of proportion of positive samples and virus concentration using z-test for two independent proportions and ANOVA, respectively.

## 3. Results 

**Figure 1:**
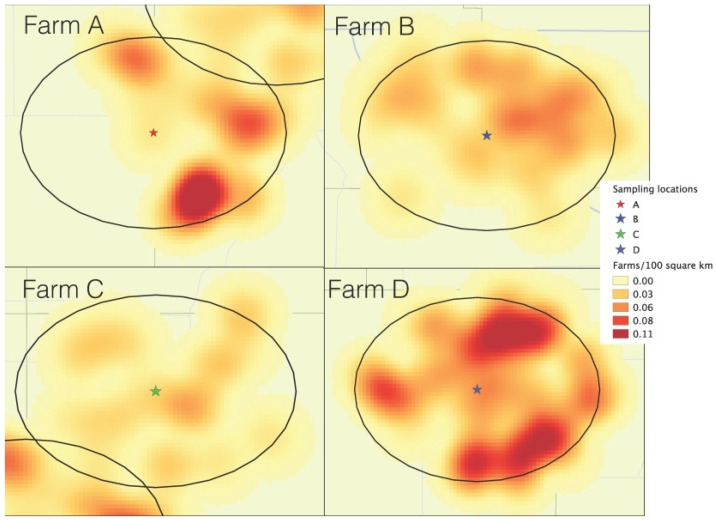
Farm density kernel within 10 km of each of the air sample locations: Farm A (South Dakota), farm B (Northeast Iowa), farm C (Southwest Minnesota), farm D (Northwest Iowa).

The description of each of the areas in terms of farm density within 10 km, and pig density within the county are shown in Table 1. The isopleth map generated using a kernel smoothing process (Figure 1) shows the density of farms within 10 km of each air sampling location. Farm C was within the lowest farm density area, including 20 neighboring farms (Table 1). Farms A and B both had 42 neighbors within 10 km, but in farm A, neighbors were clustered in half of the 10 km circle area, in contrast with farm D, which had its neighbors homogenously distributed throughout all the 10 km radius. Farm D was evidently the one within the higher farm-density-area, having 99 neighbors total throughout all surroundings within the 10 km radius. Although location A was within a higher farm‑density area than location C, the county-level pig density of location C was 17 times higher than location A (Table 1).

**Table 1:** Summary of virus collection frequency, concentration, and strain diversity in each of the air sampling collection sites.

Farm	Number neighboring farms (10 km)	County density (pigs/km^2^)	Mean (SD) distance of the farms within 10 km	Frequency (+) samples (%)	Mean viable virus in log_10_ TCID_50_/mL (range)	Number of virus sequenced	Number of virus strains *
A	42	13	7.53 (1.33)	15/51 (0.29)	4.1 (1.0–5.6)	14	9
B	42	163	6.39 (2.27)	22/53 (0.42)	4.4 (2.0–5.0)	12	9
C	20	221	6.19 (2.46)	18/54 (0.33)	3.8 (1.0–5.3)	16	8
D	99	477	6.95 (2.17)	25/59 (0.42)	5.3 (3.6–6.5)	25	14

**Figure 2:**
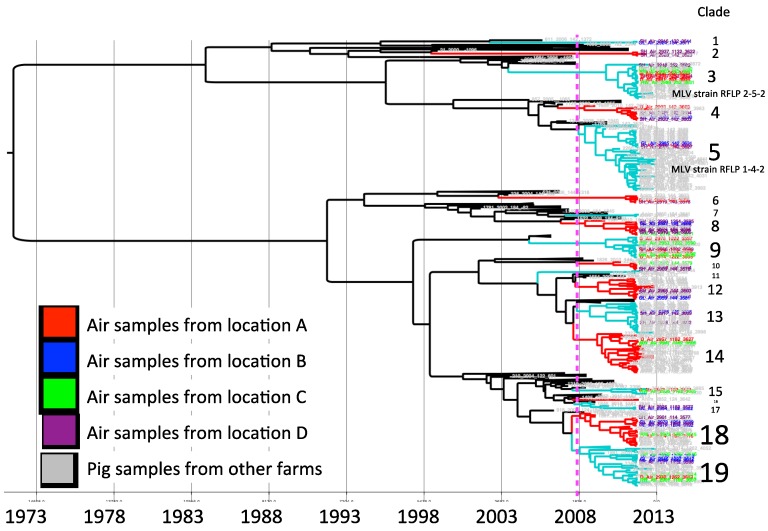
Maximum clade credibility tree. Sequences collected from air samples locations are indicated by color, location A (red), B (blue), C (green) and D (purple). The dotted line represents the cutoff to determine phylogenetic clades.

* Strains defined by phylogenetic tree in Figure 2.

### 3.1. Air Samples

Across all four sampling areas 37% (80/217) of the air samples collected were PCR positive, with frequency of positive samples in each area ranging from 29% to 42% (Table 1). When analyzed by z‑test comparison of independent proportions, the differences of the number of PRRSV PCR-positive air samples across the four sampling areas were not statistically significant (*p* > 0.05). The mean quantity of viable PRRSV in air samples ranged from 10^3.8^ to 10^5.3^ TCID_50_/mL. When analyzed by ANOVA, the mean viral titer detected in air samples around the farm D was statistically different than those observed across the other three sites (*p* < 0.01). Pertaining to quality control, all samples from collection kits prior to farm shipment and upon returning from farms were PCR-negative. All samples collected from cyclonic collectors following sanitation at the end of the sampling day across all four sites were PCR negative. In regards to air sample quality control, all repeat testing of samples by PCR (n = 80) and sequencing (n = 20) indicated identical results as the initial testing. No antibodies against PRRSV were detected in any samples, suggesting that contamination of samples during collection and/or processing did not take place. Finally, all samples (n = 30) collected from the farm site free of swine were negative for PRRSV by PCR.

### 3.2. Phylogenetic Analysis

A total of 68 air samples were sequenced and used in the analysis, of which 14, 12, 16 and 25 were from sampling locations A, B, C and D, respectively.

For model selection, the strict clock was ruled out. Based on the lowest AICM the relaxed uncorrelated exponential clock with a constant population size tree prior was selected. The MCC phylogenetic tree constructed with the selected model is shown in Figure 2. The sequences collected for the study were clustered in 19 clades according to the criteria of having a common ancestor within the five years prior to the study period. The mean percentage of paired sequence identity within each of these clusters ranged between 94.5% and 98.9%.

Clade 3 and 5 correspond to sequences closely related to modified live virus (MLV) variants, associated with RFLP 2-5-2 (MLV vaccine) and 1-4-2 (ATP vaccine). Vaccine-related sequences were detected in air samples from all four air-sampling locations.

In each sampling location, different viruses were collected. Sequences from sampling locations A, B, C and D were distributed in different clusters, some of them closely related to sequences obtained from pig samples in different clades (Figure 2). In locations A, B, and C we found similar number of viral strains as determined by a different clade (nine, nine, and eight, respectively), whereas there was a higher viral diversity in air samples from location D (14 different strains).

### 3.3. Comparison of Pairwise Sequence Identity and Genetic Distance in the Phylogenetic Tree

**Figure 3:**
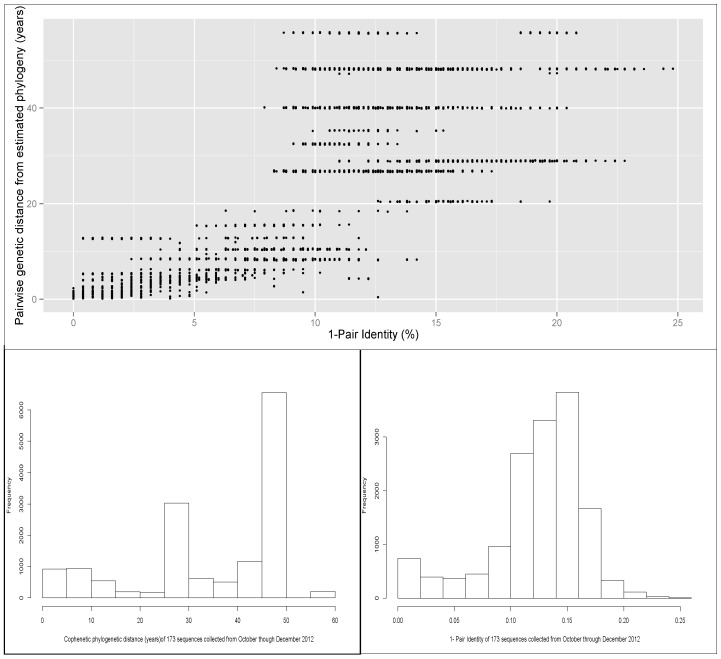
(**A**) Scatter plot of pairwise genetic identity and the distance computed from the phylogenetic tree. The total number of pairs of the 173 sequences was 14,878. Pearson’s correlation was 0.70 (95%CI 0.71-0.69). (**B**) and (**C**) Histograms of distribution of pairwise identity and tree cophenetic distance values between pairs of sequences.

A scatter plot of the pairwise identity of the 173 sequences included in the phylogeny (air samples and animal samples), and the distance calculated from the phylogenetic tree is shown in Figure 3. There was a moderate correlation (R = 0.7) between the percentage of identity and cophenetic distance between each pair of sequences. That finding suggests that there was a discrepancy in some pairs of sequences in which the distance computed by percentage of identity and the phylogenetic relationship (cophenetic distance computed from the estimated phylogeny), *i.e.*, sequences with high percentage of identity, but for which the evolutionary distance was relatively high.

The distribution of the genetic distance measured by the estimated phylogenetic tree shows that approximately half of the pair distances were between 0 and 40 years (sum of time of evolution to the most recent common ancestor), and half of the sequence pairs had a genetic distance between 45 and 50 years. The cophenetic distance between a pair of sequences in this phylogenetic tree can be interpreted approximately as twice the value estimated to reach the common ancestor (because sampling of sequences was done within a narrow window of time relative to the total evolutionary time), for example, if a pair of sequences have a cophenetic distance value of 50, then the time to the most recent common ancestor of that pair of sequences would be ~25 years. In contrast, the histogram of the distribution of pair identities showed values centered between 85% and 90% identity (Figure 3). These values in the center of the paired identity distribution account for most of the genetic distance classified differently by the cophenetic distance method, as seen in the correlation matrix.

## 4. Discussion 

This study was conducted in an effort to estimate the dynamics of airborne challenge in areas with a high density of swine production. For this purpose, we evaluated the frequency of detection, concentration and diversity of PRRSV in air samples collected around four commercial swine farms. Our results showed that in all four locations, diverse populations of PRRSV variants were present in the air, from eight to 14 different strains in one location. In addition, as hypothesized, as the density of swine production increased in a region, the frequency of detection, and concentration of virus in the air was higher. Therefore, the results of this study document that the PRRSV airborne challenge to farms in regions of concentrated swine production is significant and emphasizes the need for developing a strategy to reduce this risk when herd biosecurity plans are being designed.

In the Midwestern US, there are numerous areas with high farm and pig density, as the one described for farm D. The study suggests a very high risk of the virus entering the farm via aerosol, specifically in this study, between October and December, when there is a higher incidence of the disease. Although less aerosolized virus was detected in location C, which had the least farm-density (but high county pig density), the concentration and frequency of virus detected is also relevant to consider as a source of infection for surrounding farms.

While some previous studies have also found that airborne transmission of PRRSV as an important route of viral spread [7,9] others have suggested that the relevant route of spread is animal movement, based on molecular epidemiology [30,31,32]. In theory, if the only route of PRRS spread were airborne, an association between the space and genetic variation would be expected, but because the virus is spread by animal movement, as well as air, evidence of this association is difficult to assess. Animal movement data and complete sequence information of PRRSV in farms within an area would help understand these dynamics and adjust for virus incoming by introduction of new animals.

Another interesting finding of the study is the numerous PRRSV strains found in the air. Even in the low-farm-density areas, at least eight different viruses were sequenced. This highlights that many PRRSV strains have the ability to spread through air. This is also important, because vaccination or acclimation of gilts account for some strains, but may not protect against already circulating, or strains recently introduced in the area. This consideration, again should be more strongly considered for those areas with higher farm and pig-density, where more virus strains may circulate exemplified by the 14 different viruses present in location D.

An interesting issue arises by the fact that MLV virus was consistently isolated from air samples. If this virus reaches by air a negative farm, or another farm, it may lead to a positive PCR or ELISA result. This finding highlight the need to sequence the virus found in a farm, and may not rule out vaccine virus in a farm that does not vaccinate.

The negative results of all controls in our study support the robustness of the results obtained. Another strength of this study is the assessment of viral diversity using two different methods: computation of the pairwise identity as well as the genetic distance determined by the phylogenetic tree constructed by Bayesian methods.

Bayesian phylogenetic analysis has been used to study and understand the evolution of RNA viruses, such as Avian Influenza, HIV, among many others [33,34,35]. These methods have been popular over distance-based methods (*i.e.*, Neighbor-Joining), to estimate phylogenies of viruses for different reasons. Distance based methods only use absolute distances between pairs of aligned sequences, which can result in an underestimation of the true genetic distance if some nucleotide sites have gone through multiple substitutions events. This feature may explain, at least in part, the moderate correlation (R = 0.7) of results obtained with both methods in the study here. Bayesian methods also allow analyzing serially sampled data (the date of collection of the sample is taken into account), so the estimation of the branches lengths represents substitution per site per time period rather than just substitution per site values. It also allows computing complex models, not subject to assumptions that may not be biologically plausible (e.g., accounting for rate of heterogeneity among sites or relaxed clock models). Finally, in contrast to distance-based methods, Bayesian analysis search for the tree that best fits the data across different potential trees, and also estimates the uncertainty of the phylogeny.

The results of our study suggest that aerosol transmission of PRRSV between farms may likely be a highly relevant route of infection in swine dense regions endemic for PRRSV. We detected several viral strains circulating in the air within swine production areas, with a distinctive high concentration in the location with the highest farm and pig-density. One interesting observation is that strains from clades 1, 2 and 17 (Figure 2) were not similar to other viruses isolated from pigs included in this study. Apparently the frequency of these strains in animals is low (or alternatively may not present in local farms), but detected by environmental air sampling.

Implementation of biosecurity measures that prevent airborne transmission of PRRSV in high density areas would not only protect from PRRSV, but there is also evidence that endemic influenza can also be transmitted through aerosol, as well as exotic devastating diseases that could affect swine production, such as foot-and-mouth disease and African swine fever [36,37].

## 5. Conclusion

In conclusion, we found concentrated virus with high frequency, as well as a high diversity of strains of PRRSV in air samples collected around commercial sow farms in swine-dense regions. Concentration, frequency and number of strains were higher in areas where farm and pig densities were higher. The study suggests that aerosolized virus may likely be an important route of transmission between farms in high-density areas. Results also suggest that computing pairwise identity between sequences pairs, as a measure of genetic distance, may not always reflect the true evolutionary distance. These results will contribute to the understanding of the dynamics of PRRSV spread and, ultimately, the prevention and control of one of the most devastating diseases of swine.
